# Imaging persistent spinal pain syndrome and spine surgery complications: an interpretation guide for radiologists

**DOI:** 10.1186/s13244-025-02065-8

**Published:** 2026-01-28

**Authors:** Jean-François Budzik, Tatiana Musset, Guillaume Lefebvre, Julie Legrand, Julien Decaudain, Vincent Ducoulombier, Sébastien Verclytte

**Affiliations:** 1 Service d’imagerie diagnostique et interventionnelle, Groupement Hospitalier de Brocéliande Atlantique, Centre Hospitalier Bretagne Atlantique, Vannes, France; 2 Service d’imagerie diagnostique et interventionnelle, Groupement des Hôpitaux de l’Institut Catholique de Lille, Hôpital Saint Philibert, Lomme, France; 3 Service de chirurgie orthopédique et rachidienne, Groupement des Hôpitaux de l’Institut Catholique de Lille, Hôpital Saint Philibert, Lomme, France; 4 Service de rhumatologie. Groupement des Hôpitaux de l’Institut Catholique de Lille, Hôpital Saint Vincent de Paul, Lille, France

**Keywords:** Spine, Postoperative pain, Failed back surgery syndrome, Imaging

## Abstract

**Abstract:**

This educational review provides a comprehensive guide for radiologists on the imaging interpretation of persistent spinal pain syndrome (PSPS) and complications following spine surgery. PSPS, previously known as failed back surgery syndrome, describes persistent or recurrent, primarily neuropathic, pain after spine surgery affecting 10–40% of patients. Radiologists often encounter challenges in diagnosing PSPS due to unfamiliarity with postoperative anatomical modifications and the complexity of surgical interventions. This review emphasises the necessity of correlating imaging findings with the clinical context through an interdisciplinary collaboration, while keeping in mind the particular psychological context of postoperative patients in chronic pain. We focus on lumbar spine surgery such as lumbar spine discectomy, lumbar spine stenosis, posterior decompression and stabilisation-fusion procedures. The review offers practical insights into managing key clinical scenarios: early complications with genuine emergencies, but also more subtle diagnoses such as low-grade infections or hardware failures. We underscore the utility of various imaging modalities—radiography, CT, MRI, PET and SPECT, and propose the ideal combination for each clinical situation. Plain radiographs are useful for assessing patients in standing positions and detecting intervertebral instability. CT is ideal for examining bone fusion and surgical hardware, while MRI excels in soft tissue analysis. PET and SPECT provide crucial insights into bone metabolism, detecting micromobility or infections. Based on 15 years of interdisciplinary collaboration, this guide, based on clinical scenarios, aims to enhance radiologists’ confidence and accuracy in interpreting postoperative spine imaging, improving diagnostic precision, patient management and communication with referring clinicians.

**Key Points:**

- Managing postoperative spine imaging is often challenging for surgeons and radiologists.
- Postoperative spine imaging requires precise clinical correlation and careful multidisciplinary evaluation.
- Different imaging modalities can be combined to answer difficult issues.

**Graphical Abstract:**

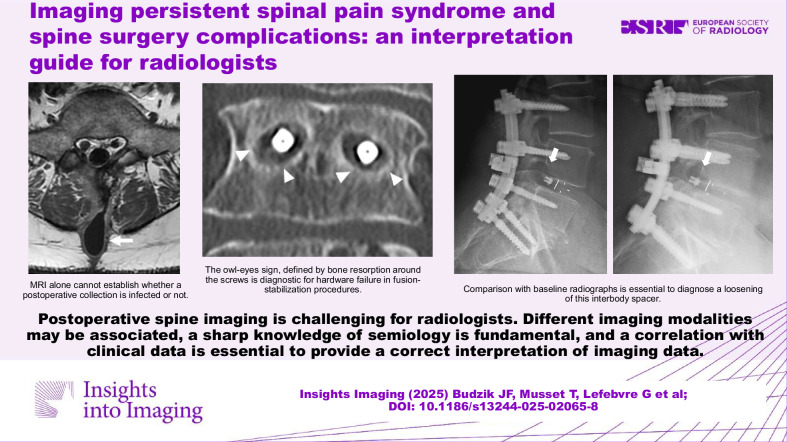

## Introduction

Failed back surgery syndrome is an umbrella term referring to painful sensations occurring after spine surgery. More precisely, it is defined as either a persistent pain or a recurring pain in a post-spine surgery context, the pain being mostly neuropathic [1]. It concerns 10–40% of back surgery patients [2]. Recently, experts advocated for replacing this term with ‘persistent spinal pain syndrome’ (PSPS) [3] or ‘chronic pain after spinal surgery’ under the new International Classification of Diseases (ICD-11), which has been accepted by the World Health Organization [4]. Indeed, the term ‘failed back surgery’ is a stigmatising term that shifts the blame onto the surgeon, setting aside the complexity of such situations, which need to be analysed thoroughly, looking for a causative anatomical or non-anatomical source of pain among a wide spectrum of possible conditions [5].

Radiologists can feel queasy when facing a patient complaining of back pain after spine surgery, as they may not be used to the modified anatomy induced by surgery and the techniques and devices used in spine surgery. Moreover, they may not understand the whole reasoning, what kind of abnormalities can be found, and how these abnormalities may present in these particular situations. Yet, a correlation considering the clinical setting is mandatory to reliably interpret imaging data [6].

Radiologists must also realise that patients suffering from chronic pain related to spine conditions have high expectations of spine surgery, which they often consider as their last chance for total pain relief. Patients may be in an emotional state of distress if they do not experience this relief. Consequently, they may be eager to know the results of their imaging investigations to understand what has gone wrong. Unsuitable or evasive conclusions may worsen the problem by affecting their psychological well-being or relationship with the referring doctors [7].

Previous research has brilliantly described the normal appearance of the postoperative spine [8] and presented the inputs of the different imaging techniques [1, 7]. These prerequisites are essential, and we invite readers to refer to these open-access articles, as we will not repeat these points. It is essential to understand the techniques, the hardware, and the normal postoperative appearance before going into pathological findings.

Indeed, the purpose of this article is to review the common clinical complaints that can require imaging assessment, to identify which diagnoses must be discussed, which examinations must be done, and the findings associated with these diagnoses. Its aim is to provide pragmatic insights into real-life clinical-based situations, issued from 15 years of close collaboration between musculoskeletal radiologists, spine surgeons and rheumatologists who meet patients during the same consultation.

Because spine surgery is a broad topic, we will focus on the more common situations, which are: the lumbar spine, discectomy, lumbar spine stenosis (LSS), posterior decompression, and stabilisation-fusion procedures involving hardware (screws, rods and cage spacers).

## Early complications: the first hours or days after surgery

Early complications are absolute or relative imaging emergencies because they require a quick therapeutic action [9]. Clinical sign descriptions are available as Supplementary Material.

### Early postoperative neurological deficit

#### Acute cauda equina syndrome

This situation requires a lumbar spine magnetic resonance imaging (MRI) (by default, a computerised tomography (CT) scan) within hours, as nerve ischaemia can lead to permanent motor deficit with paraplegia.

**Fig. 1:**
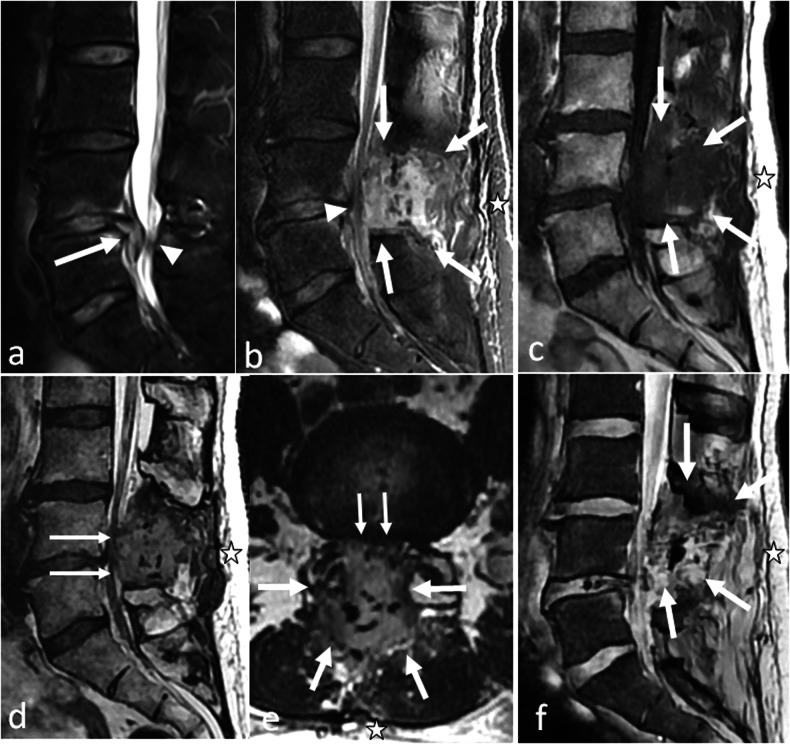
Cauda equina syndrome occurring three hours after a L4–L5 discectomy in 56 years old patient. MRI was performed in an emergency setting. On preoperative fat-suppressed sagittal T2-weighted images, a disc herniation issued from L4–L5 disk (arrow) was responsible for a dural sac compression with persistent cerebrospinal fluid (arrowhead) (**a**). On postoperative fat-suppressed sagittal T2-weighted images (**b**), sagittal T1-weighted images (**c**), sagittal (**d**) and axial (**e**) T2-weighted images, a heterogeneous collection lies in the operating bed (arrow), which results in a severe narrowing of the dural sac (arrowhead) with no persistent cerebrospinal fluid; this is best seen on axial images. Note that this is a deep haematoma without superficial component, thus no external bleeding (asterisks). Also, the dark elements that are visible within the haematoma on T2-weighted images (**b**, **d**, **e**) correspond to biological glue (**f**). On gradient echo T2-weighted images, there is no significant signal drop as this is an acute haematoma without hemosiderin. This latter sequence is not mandatory, and should not delay surgical re-intervention

An epidural spinal haematoma is an extra-dural collection resulting in dural sac compression, without visible cerebrospinal fluid around the roots. The signal of this collection is variable, but it is expected to be heterogeneous, with a dominant hyperintensity on T1-weighted sequences (persisting after fat suppression) and T2-weighted sequences (Fig. 1).

#### Acute medullary syndrome

**Fig. 2:**
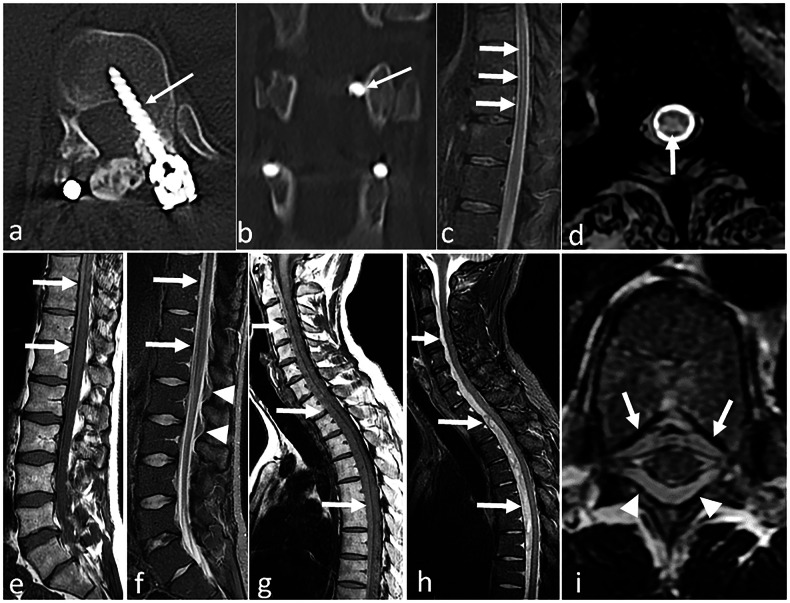
**a**, **b** Acute medullary syndrome in a patient waking up from anaesthesia after a thoracolumbar arthrodesis. On CT-scan the left D12 screw is in the spinal canal (arrow) on axial (**a**) and coronal (**b**) reconstructions. **c**, **d** Acute spinal cord ischaemia in another patient. MRI was performed for a flaccid paraplegia with saddle anaesthesia occurring immediately after herniation treatment. It shows high T2 signal within the cord (arrows) on sagittal (**c**) and axial (**d**) views. **e**–**i** Epidural haematoma in a third patient. An acute paraplegia was diagnosed following lumbar disc surgery. MRI shows epidural anterior collections that are hyperintense on both T1- and T2-weighted images (arrows). Note that they are difficult to identify on lumbar acquisitions (**e**, **f**). Collections appear more clearly on thoracic acquisition on sagittal T1 (**g**) and fat-suppressed sagittal T2-weighted images (**h**), as well as on axial T2 images, where a posterior component is also identified (arrowheads) (**i**)

Intuitively, spinal cord compression should not be a complication of a lumbar spine surgery. Yet no anatomical obstacle impedes the caudo-cranial diffusion of haematomas in the epidural space: postoperative spinal cord compression is a warning sign of an epidural haematoma. It can also result from an iatrogenic lesion, either preoperative, or related to misplaced hardware (Fig. 2). This situation requires an urgent full spine MRI.

Consequently, in case of a neurological deficit occurring after spine surgery, if the clinical presentation is unclear, or if the interpretation of the lumbar spine is equivocal, MR imaging should not be restricted to the lumbar spine and include a full spine acquisition (Fig. 2).

#### Conus terminalis infarction

This can occur by an iatrogenic occlusion of the Adamkiewicz artery, which can be found between T8 and L3, mainly (two-thirds) on the left side, and in one in two cases, in the L1–L2 or L2–L3 foramens.

MRI is the modality of choice. The main features are hyperintensity of the conus terminalis on T2-weighted images with restricted diffusion on diffusion-weighted imaging (Fig. 2).

### Blood loss

Postoperative blood loss requires an urgent CT exam, including non-contrast and post-contrast acquisitions. Arterial- and portal-phase acquisitions are mandatory. A supplementary late acquisition can be added if the interpretation of previous acquisitions is equivocal: suspected bleeding without clear extravasation. The degree of emergency depends on the tolerance of the patient: it is an absolute emergency in case in the setting of haemorrhagic shock. Intra- or retro-peritoneal haematomas are not subtle findings (Supplementary Fig. 1). Collections in the surgical bed are common findings, and their volume must be considered together with the extent of the bleeding. Radiologists must pay attention to subcutaneous tissues, as venous bleeding can appear as a hypodense infiltration in the back area. This should not be confused with oedema, which can be found in patients suffering from hydrops, as the distribution of subcutaneous abnormalities is different, and also because the context is different: these patients have other fluid effusions (pleural, peritoneal, leg, or upper back subcutaneous tissue) (Supplementary Fig. 1).

### New radicular pain

If a patient complains of new radicular pain after recovering from anaesthesia, an iatrogenic nerve root lesion must be sought first.

CT scan is the examination of choice. Identifying an impingement between a screw and a nerve root can be straightforward, and comparing left and right structures is often enough to establish the diagnosis (Supplementary Fig. 2). Yet, beam hardening artefacts can make it difficult to identify the nerve root precisely. If an MRI is performed, it is important to know that even if susceptibility artefacts may not seem important, MR never allows direct visualisation of surgical hardware: CT and/or X-rays must be performed to determine any conflict between a nerve root and a surgical device (Supplementary Fig. 2). Impingement with an intersomatic cage is also possible. Oversized hardware can be the cause (Supplementary Fig. 2). One must be careful not to overdescribe potential impingements if the cage comes close to the foramina, especially in fusion procedures with transforaminal approaches where the cage is not centred in the intersomatic space [8].

Nerve roots can also be wounded during surgery, either directly by a screw that will be repositioned thereafter, or indirectly via ischaemic phenomena, in what has been named ‘battered nerve root syndrome’ [1]. In this latter situation, prolonged contact with a surgical instrument (as in nerve retractions in disc surgery) may impede the microvascularisation of the root. Consequently, in the absence of surgical hardware impingement (non-contributory CT), MRI has to be performed, with and without IV contrast. A battered nerve may appear swollen, hyperintense on T2-weighted images and enhance intensely after contrast media injection (Supplementary Fig. 3). This must be distinguished from the normal epidural scar enhancement that can be encountered around a nerve in disc surgery.

One must not forget that postoperative leg pain can have other causes, such as deep vein thrombosis (Supplementary Fig. 3). This condition can be secondary to local compression by misplaced hardware, but it is more frequently related to the postoperative context.

### Infection

**Fig. 3:**
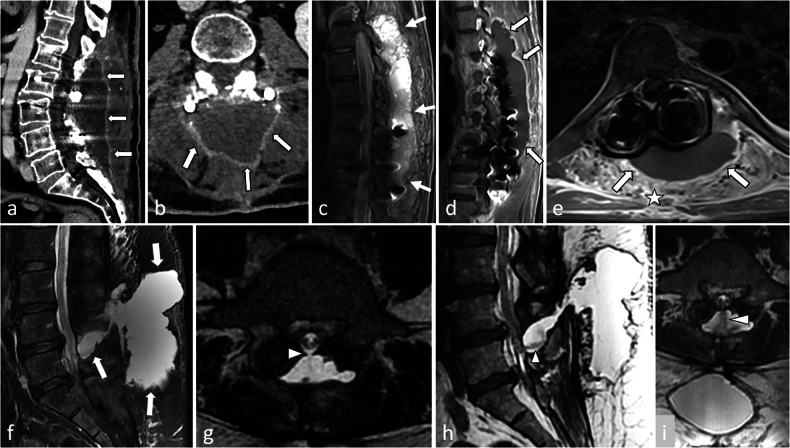
**a**, **b** Post-contrast CT-scan performed 5 days after a L1–S1 arthrodesis in a patient with fever, inflammatory scar and purulent drainage. Large posterior fluid collection (arrows) with peripheral enhancement in close contact with the surgical hardware on sagittal (**a**) and axial (**b**) reconstructions, corresponding to an abscess. c–e Another patient with signs of local infection in the days after a thoraco-lumbar arthrodesis. MRI shows a large fluid collection of the surgical site (arrows) on sagittal fat-suppressed T2-weighted sequence (**c**), and on sagittal (**d**) and axial (**e**) fat-suppressed post-contrast T1 sequences, corresponding to an abscess. Note the diffuse enhancement of muscles in the surgical bed (asterisk), that is not specific of infection in an early post-operative context. **f**–**i** MRI performed for a postoperative headache in a patient operated of a disc herniation. On sagittal fat-suppressed T2-weighted images (**f**) a large posterior fluid collection of the surgical bed (arrows) is in close contact with the dural sac. High-resolution (isotropic voxel of 0.6 mm) T2-weighted images (**g**) shows the communication with the intra-dural space through a dural tear (arrowheads). A flow artefact of intermediate signal (arrowheads) corresponding to the leak of cerebrospinal fluid is also visible on sagittal (**h**) and axial (**i**) high resolution T2-weighted images

MRI with and without IV contrast is the modality of choice [10]. The topography of the abnormalities depends on the type of surgery: in acute infections, manifesting with fever and elevated blood markers in the days after surgery, the bacterial graft infection is expected in the surgical bed, and pyogenic germs are involved (Fig. 3). This is different from haematogenic spine infections that occur in non-operated patients. This is also different from low-grade infections that will be discussed later on.

**Fig. 4:**
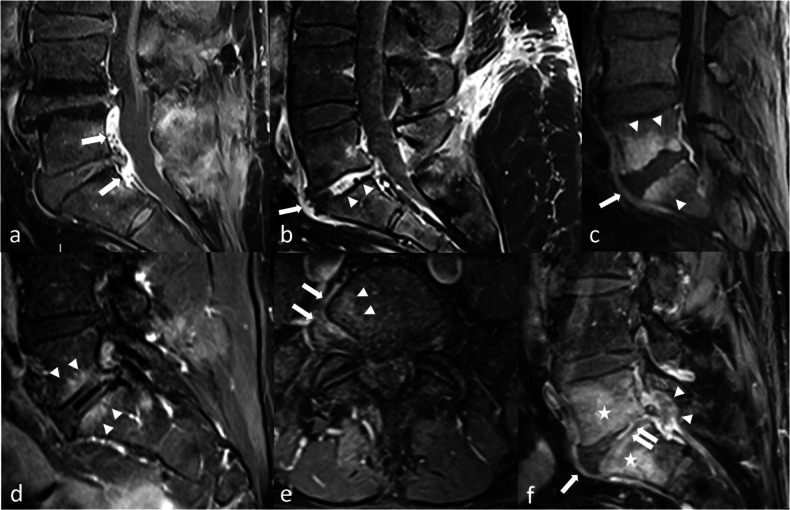
**a** Normal enhancement of the anterior epidural space (white arrows) after disc surgery on post-contrast fat-suppressed sagittal T1-weighted MRI images. **b** In this other patient with mild postoperative back pain after disc herniation removal, post-contrast fat-suppressed sagittal T1-weighted MRI images showed an expected disk enhancement (arrowheads), but also an atypical pre-vertebral enhancement (arrow). After negative biology and close follow-up, infection was ruled out and the patient recovered. **c** Classic aspect of haematogenic L5–S1 spondylodiscitis with oedema and extensive enhancement of both vertebral end-plates (arrowheads) and prevertebral tissues (arrow) on MRI fat-suppressed sagittal T1-weighted images (**c**). **d**–**f** Another patient benefited from L5-S1 laminectomy and intra-canalar facet joint cyst removal. MRI was performed because of mild postoperative lumbar pain associated with mild inflammatory syndrome. On sagittal (**d**) and axial (**e**) post-contrast fat-suppressed T1-weighted images, lateralised peri-discal oedema (arrowheads) extending in the adjacent soft tissues (arrows) was neglected, although this was atypical as these structures were not supposed to be concerned by surgery. The patient got worse and another MRI revealed features of spondylodiscitis with extensive peri-discal oedema (asterisks), and prevertebral (arrow), disk (double arrows), and foramino-epidural (arrowheads) enhancement: post-contrast fat-suppressed T1-weighted images (**f**)

As with a spine not undergoing surgery, identifying an abscess is the cornerstone of the diagnosis (Fig. 3). They are located in the surgical bed [7]. However, in a postoperative setting, image interpretation is challenging because some morphological alterations are common findings, particularly soft tissue (and often bone) oedema [10] (Fig. 4). Other fluid collections, such as haematomas and seromas, are also frequent in the surgical bed, most of which do not require any treatment. If their borders are not convex, this means that they may not be under pressure, which can be an indirect negative sign of infection (Supplementary Fig. 4). However, these collections may become infected. It is important to keep in mind that neither CT nor MRI can determine for sure whether a postoperative collection is sterile or infected. Peripheral contrast enhancement is not discriminant in an early postoperative context, as it is commonly encountered around seromas (Supplementary Fig. 4). The analysis of the fluid is not helpful either: although the semiology of haematomas, seromas and abscesses is often reported in the literature [11], in our experience, signal or density analysis is rarely a game changer, as many reported differences are theoretical.

Thus, if the question of a local infection arises, the role of the radiologist is to determine whether there is a significant fluid collection in the surgical bed. If so, a supplementary needle aspiration must be performed under local anaesthesia. Needle diameter should not be smaller than 19 gauge, as fluid can be thick, particularly in cases of haematoma with a secondary infection. Microbiological analysis is the only way to establish whether a fluid collection contains bacteria or not.

During surgery, dura mater tears can be repaired by a patch sprayed with fibrin glue. Although the patch itself is not visible, the glue can be identified on MRI as a hypointense avascular structure on all sequences, amid the inflammatory postsurgical scar. This should not be misinterpreted as gas but as an anaerobic abscess (Supplementary Fig. 4). Talking with the surgeon or reviewing the surgical report is, of course, extremely helpful.

In case of bone graft, the harvesting site, which is usually in the posterior aspect of one (or both) iliac bone(s), should not be mistaken for a bone abscess, as it associates bone ‘erosion’ and gas bubbles, which correspond to air trapped within haemostatic sponges, such as Surgicel© devices (Supplementary Fig. 4).

### Headache

Early postoperative headache is suggestive of intracranial hypotension secondary to a pseudomeningocele [9], which represents a fluid collection of cerebrospinal fluid that communicates with the intradural space but is not confined by dura mater or a surrounding membrane [6]. Unlike meningoceles, the borders of a pseudomeningocele consist of reactive fibrous tissue [7]. Headaches are usually severe, with positional features, typically worsening when standing and sitting upright, and also when bending the head forward, while relief occurs when lying down. Accompanying symptoms may include nausea, vomiting, dizziness, light-headedness, tinnitus, and blurred vision.

MRI is the best modality to search for a pseudomeningocele, which is a fluid collection in close contact with the thecal sac, and resembles cerebrospinal fluid on all sequences, without significant enhancement (Fig. 3). We suggest performing high-resolution T2 images to try to identify the source of the leak, in close contact with the thecal tear (Fig. 3), the location of which should be described in the surgical report. With larger tears, a cerebrospinal fluid flow artefact can be seen on T2-weighted images between the dural sac and the pseudomeningocele, which may not be visible on fat-suppressed T2-weighted images (Fig. 3). If the communication between the fluid collection and the dural sac is not seen, and if diagnosis is unclear, it may be helpful to inject iodinated contrast medium into the subdural space [7].

## Treatment inefficacy: pain continues after surgery

In such cases, the patient often leaves the hospital as no major complications are detected. Yet the patient is dissatisfied with the treatment as they are still experiencing pain.

Regarding the notion of pain-free interval, see Supplementary Material.

### No change in pain

If the patient reports no improvement in pain, the radiologist must determine whether the cause of the pain has been correctly treated.

#### Level errors

If the patient is not relieved at all, one must ensure that the surgery addressed the correct spine levels.

Whether in LSS decompression or in herniectomy procedures, one can assess whether the surgical path (fascial and muscular scars, laminectomy) corresponds to the targeted level, in comparison with the preoperative imaging (Supplementary Fig. 5). After a discectomy with a level error, the appearance of the herniated disc is exactly the same as before, and the surgical scar is located at another level (Supplementary Fig. 5).

In our experience, level errors are often caused by miscommunication between the radiologist and the spine surgeon, the denomination of the lumbosacral transitional variant being the main problem, when radiologists and surgeons do not label the last vertebras in the same way.

Always keep in mind that only C1 and C2 can be named with certainty because of their particular morphology [12]. As many others [12], we believe that despite classification systems and old concepts (‘lumbarization’ or ‘sacralization’ with their ‘hemi-’ variants), nothing replaces an explicit description of the anatomical features of this lumbosacral junction.

We advise radiologists to clearly mention any transitional variant from the beginning of their report, describing this variant, and explicitly naming the vertebras according to this description, whatever the modality. A key image is extremely useful to the surgeon and serves as a reference for intraoperative fluoroscopy [8].

We advise spine surgeons to read radiologists’ reports. Surgeons might not agree with the radiologists’ vertebral numeration, but this is of no consequence as long as they are aware of this particular anatomy.

#### Insufficient dural sac decompression in LSS

After LSS decompression, CSF must return around the roots at every level of the dural sac. MR is the modality of choice as CT cannot discriminate the nerve roots in the dural sac.

Incomplete decompressions resemble a persistent stenosis that is grade C or D according to Schizas et al [13]. Some of the extrinsic structures responsible for the compression are still present: osteophytes in the spinal canal, ligamentum flavum or disc bulging (Supplementary Fig. 5).

If the dural sac decompression looks correct but symptoms have not improved, one must consider an associated dynamic stenosis, secondary to intervertebral instability. This can be diagnosed on plain radiographs with upright profile incidences in a neutral position, maximal flexion and maximal extension [14].

#### Technically perfect treatment, but no improvement

This paragraph is available within the Supplementary Material.

### Change in pain: Low-grade infection?

In addition to acute infections that are clinically apparent, with severe pain, fever, and abscess formation (discussed previously), low-grade infections can occur, and be far more difficult to diagnose. The main symptom is a change in pain, or the occurrence of ‘supplementary’ pain, which tends to be dull and constant, with possible inflammatory features (waking or morning stiffness). Such a change may not be clearly identified by the patient, and thus by his/her doctors, resulting in late diagnosis. Fever may be present. The lab work may not be demonstrative: blood markers like C-reactive protein are frequently subnormal (mild elevation) and can even be normal.

MRI with and without IV contrast is the modality of choice [10, 15]. Imaging features vary according to the site of the bacterial graft infection. CT must be performed if the interpretation of the MRI is equivocal.

**Fig. 5:**
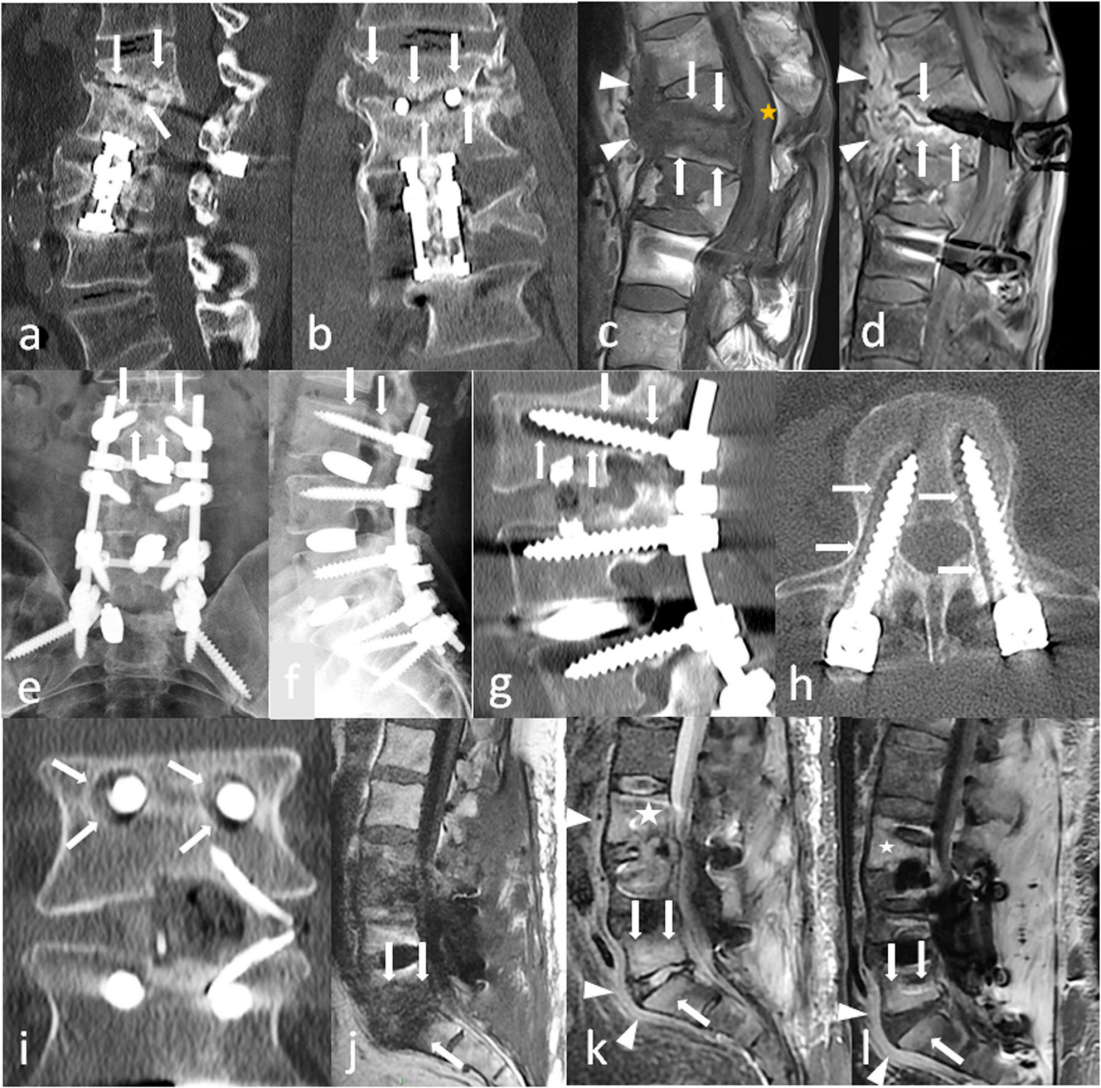
**a**–**d** Patient follow-up few months after a T12–L2 fusion procedure for a L1 split fracture with persistent back pain and inflammatory syndrome. Sagittal (**a**) and coronal (**b**) CT reconstructions show a T11–T12 large vertebral body resorption with irregular margins and associated sclerosis (arrows) with kyphotic deformation. On coronal images, T12 screws are within the disk. On complimentary MRI with sagittal T1- (**c**) and post-contrast fat-suppressed T1 images (**d**), oedema (arrows) of T11 and T12 bodies and frankly enhancing prevertebral soft tissue thickening (arrowheads) confirm the diagnostic of postoperative spondylodiscitis with T11 body resorption. **e**–**i** A different patient complained of persistent pain 6 months after a posterior lumbo-sacral arthrodesis. On anteroposterior (**e**) and lateral (**f**) X-rays, and on sagittal (**g**), axial (**d**) and coronal (**h**) CT reconstruction, an extensive osteolysis is visible around the L3 screws with no sclerotic rim (arrows) and typical « owl eyes » on coronal reconstruction (**i**) that should suggest septic pseudarthrosis. Note the absence of equivalent abnormality around the L4 screws. **j**–**l** This other patient suffered from post-operative back pain with inflammatory syndrome after an L3–S1 fusion procedure. MR images reveal extended peridiscal oedema in L5–S1 (arrows), with prevertebral inflammation (arrowheads), which correspond to a spondylodiscitis. Oedema in L3 vertebral body (asterisk), raised suspicion of extended infection in this context, especially since prevertebral tissue show inflammation (arrowhead). Yet one must keep in mind that the meaning of vertebral body oedema alone is debatable

In disc surgery, contrary to haematogenic spondylodiscitis, in which prevertebral soft tissue inflammation is the cornerstone of diagnosis, followed by oedema of the anterior corners of the vertebral body (Fig. 4), postoperative spondylodiscitis begins in the posterior aspect of the disc margins. However, genuine infection must be distinguished from common postoperative changes corresponding to the mechanical irritation of the vertebral endplate or the epidural space, for example in the treatment of disc herniation (Fig. 4). In the latter, postoperative changes may appear as posterolateral oedema of the vertebral endplates, on one or both sides of the operated disc, which stays close to the vertebral endplates. Small erosions can be present. After several weeks, these erosions will be surrounded by a thin sclerotic rim corresponding to bone healing. On the contrary, septic erosions have irregular contours. Surrounding bone is normal in the first stages of infection, but irregular sclerosis can be present if it becomes chronic. Identifying abscesses in the vertebral bodies, anterior epidural space or surrounding soft tissue is a game changer, as it is highly suggestive of infection [15]. Despite these theoretical differences, the distinction between postoperative changes and low-grade infection can be very difficult to establish. MR and CT follow-up may be helpful: a progression of bone and soft tissue oedema and erosions will be highly suggestive of creeping infection (Fig. 5).

In fusion surgery, infection around the screws results in irregular, ill-defined margin osteolysis extending away from the bone-hardware interface (Fig. 5). Inversely, limited and regular circumferential osteolysis centred on the screw, with regular margins and a thin sclerotic rim, is suggestive of mechanical loosening. In ambiguous cases with inconclusive MRIs, nuclear medicine techniques can be the game changer: at first, FDG-PET/CT, to detect abnormal bone metabolic activity, and then a gallium scan, which is more specific for infection [7, 10]. Labelled leucocyte SPECT-CT has fallen out of favour in this indication [16].

Discovertebral biopsy are often required to identify the causal germ, as sepsis does not occur in chronic low-grade infections.

## Late complications: pain recurrence

This is the most frequent situation in spine clinics. After a genuine period of relief (weeks, months or years), the patient complains of pain recurrence.

### Clinical foreword

A thorough anamnesis is key to understanding the situation. Indeed, as in spine complaints, imaging interpretation relies on clinical hypothesis. The pain-free interval must be analysed to determine whether it is real (the subject of this chapter), with a positive impact on everyday activities, or if the improvement was transient when the patient stayed still and/or took stronger pain medication in the days after surgery. Also, one needs to decipher the complaint to ascertain whether it is a recurrence of pain (the same symptoms as before surgery) or another pain (and therefore possibly unrelated to the surgery). This can be tricky, especially in patients with chronic pain and intricate sources of pain.

### Radicular pain

#### Disc herniation recurrence vs epidural fibrosis

##### Clinical foreword.

The question of a disc herniation recurrence may be very difficult to answer. First, the hernial image may be present up to 6 months after surgery [9]. Second, asymptomatic recurrent disc herniation has been reported in up to 1 in 4 patients two years after surgery [17]. Last, active changes occur within the epidural scar, maturing for at least 6 months after surgery, making it potentially very difficult to identify a disc herniation recurrence within the scar.

The possibility of herniation may be questioned if the patient reports pain in the same radicular territory, after a genuine pain-free interval. Indeed, if the patient reports pain in the other side, or in another sensitive territory on the same side, then the surgical bed is likely not the problem: such a situation can be analysed as in patients not undergoing surgery. The clinical theoretical distinction between both entities relies on the progressive onset of pain with neuropathic features in epidural scars vs rapid onset pain with impingement signs (such as in the straight leg raise test).

##### Theoretical distinction.

An epidural scar is in continuation with the surgical approach that is visible in the paravertebral muscles, through the ligamentum flavum, along the lateral epidural space, up to the disc. It can include the nerve root. Its signal is usually slightly higher than the disc on T2-weighted images [15] (Supplementary Fig. 6). In the first months after surgery, inflammation is active in the surgical path, so the scar may be swollen, with a slight mass effect on the dural sac, which appears isointense on T1-weighted MRI and hyperintense on T2-weighted MRI and clearly enhanced on T1-weighted fat-suppressed MRI (Supplementary Fig. 6). After several months, it becomes retractile, attracts the dural sac, and may enlarge the nerve root (Supplementary Fig. 6). Enhancement decreases then disappears.

**Fig. 6:**
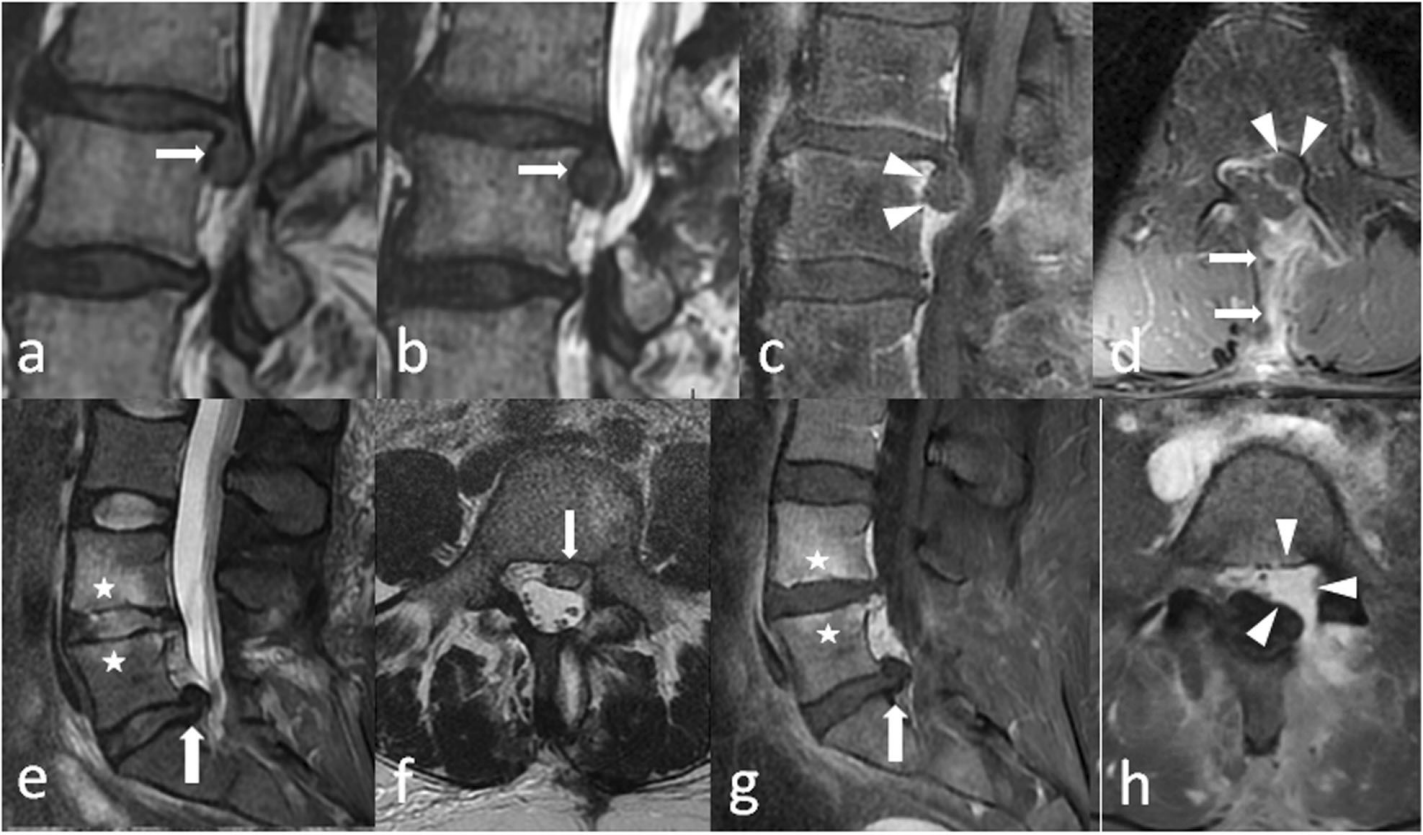
**a**–**d** Recurrent radicular pain in the same territory 1 month after surgery. Preoperative (**a**) and postoperative (**b**) sagittal T2-weightd images look alike, with a slighltly more prominent herniation in b, which made it suspicious of hernia recurrence. On sagittal (**c**), and axial (**d**) post-contrast images, this surgical bed enhances (arrows), but the herniation does not, which is diagnostic for hernia recurrence. **e**–**h** This other patient complains of recurrent pain in the same radicular territory. On preoperative sagittal T2-weighted images (**e**), a L5–S1 disc herniation was present (arrow). On postoperative axial T2-weighted images (**f**), a nodular hyperintense image is present in the surgical bed, in contact with the disk, which appears at first as a hernia recurrence. On post-contrast images, the image does not enhance on the first sagittal acquisition (arrow) (**g**), which is evocative of recurrence. Yet, on the second axial acquisition (**h**), the anterior and left epidural space enhances (arrowheads), which traduces the diffusion of the contrast media in the scar. Disc herniation was therefore excluded. Note a L4–L5 discopathy with peridiscal oedema (stars) that was already visible before surgery (**e**)

Inversely, recurrence of a disc herniation is in continuation with the disc, and is expected to have the same signal as the disc. It creates a mass effect on the adjacent root(s) and the dural sac, and does not clearly enhance after gadolinium injection [15] (Fig. 6).

Thus, high-resolution sequences such as 3D isotropic T2-weighted sequences are fundamental to differentiate epidural scarring from hernia recurrence [15]. Gadolinium injections can help by distinguishing a fully enhancing scar from a peripheral-enhancing herniation (Fig. 6).

Although these diagnoses are theoretically different, frontiers can be blurred, making this distinction one of the greatest challenges of post-therapeutic spine imaging. The signal of a genuine herniation on T2-weighted images can be increased and mistaken for epidural scarring. Additionally, the disc herniation can be enhanced moderately by passive diffusion [15].

##### Back to basics.

The possibility of hernia recurrence is a challenge for most radiologists, who manage the issue with systematic gadolinium injections. Yet, talking to the patient, and referring to preoperative images is enough to manage most patients without contrast.

First, if no disc herniation is seen, it is not a recurrence.

Then, if a herniation is present in the surgical bed, it should be compared with the preoperative images. If the current herniation has different features (e.g., larger, ascending instead of descending, or vice-versa), it is obviously a recurrence. In these situations, the morphological analysis on 3D T2 images is sufficient, and contrast injection is unnecessary (Supplementary Fig. 6).

Post-contrast images must be performed if preoperative and postoperative images are similar (Fig. 6). In this case, contrast will be the game changer, as the postoperative scar will enhance all the way along, up to the contour of the disc or even within. Conversely, if a disc herniation is present within this scar, it will not enhance as the disc is far less vascularised than the epidural space (Fig. 6). Thus, gadolinium can help but is generally not useful more than 6 months after surgery [18], as mature scar is not expected to enhance after contrast injection.

**Fig. 7:**
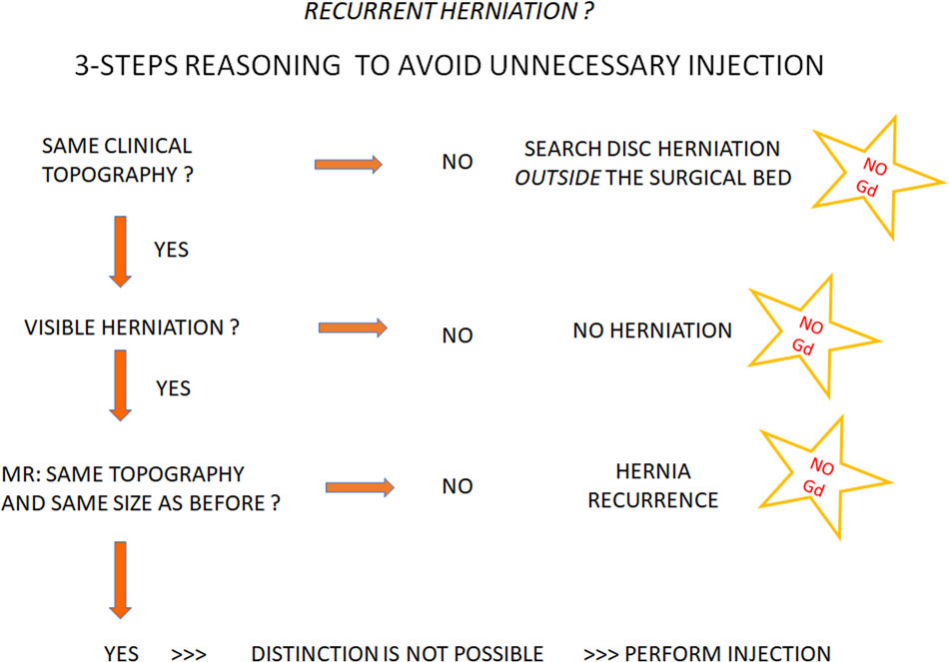
In-house clinically-based logigram used to decide whether gadolinium should be injected or not in patient who benefited from disk surgery in the precedent year. Gd, gadolinium

Thus, in contrast to articles written by non-radiologists [1], we do not perform systematic gadolinium injections due to safety, environmental and economic concerns. Instead, we refer to our custom algorithm to decide whether or not contrast agents should be injected (Fig. 7).

Lastly, in our opinion, the term ‘epidural fibrosis’ should be avoided as it suggests both an established situation and a cause for symptoms. In actual fact, fibrosis is a dynamic process (active during maturation, then inert), and its responsibility in pain symptoms is subject to caution [1, 19]. In our facility, we prefer to use ‘epidural scar’, which we consider as more neutral.

#### Arachnoiditis

**Fig. 8:**
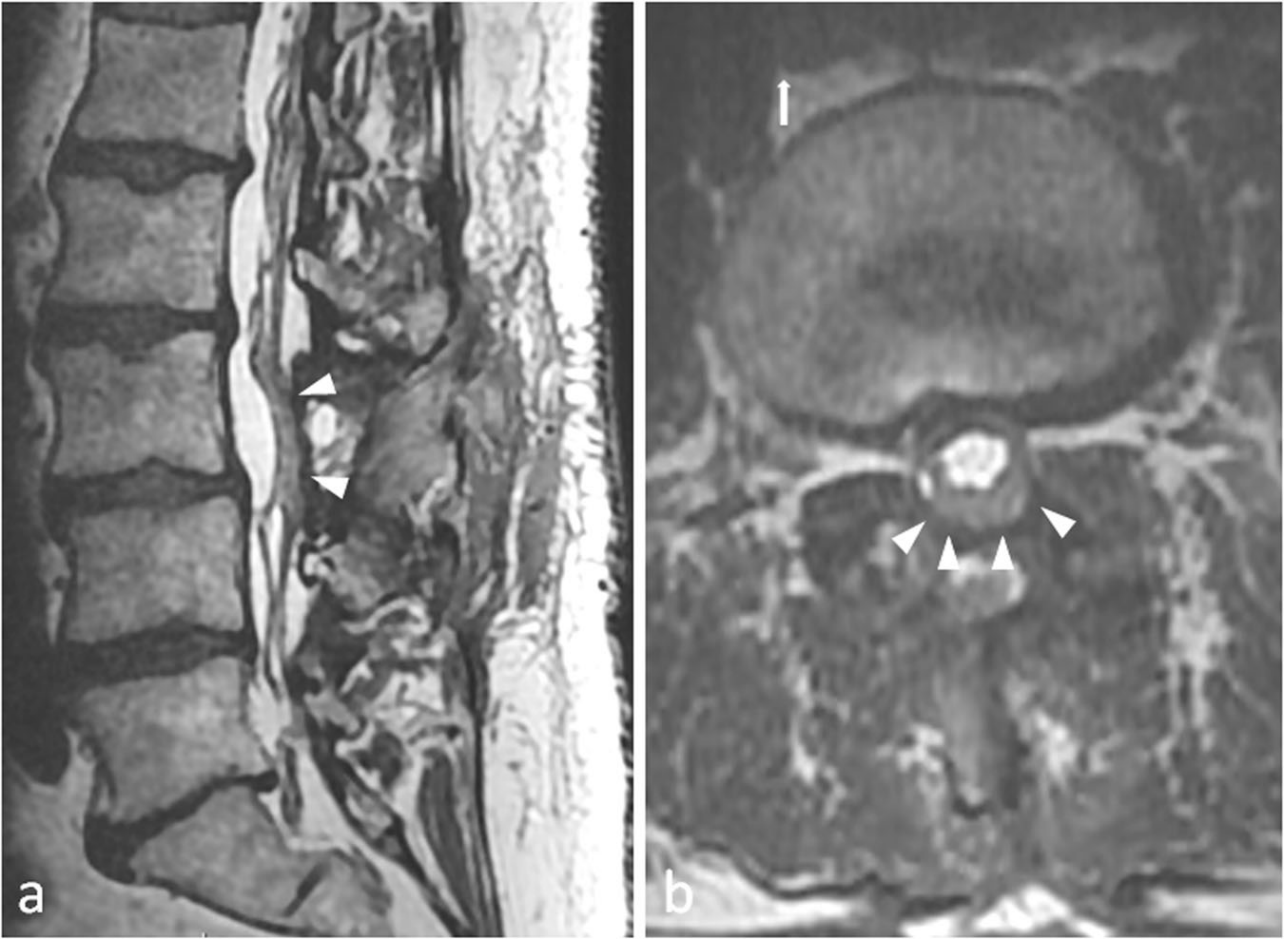
This patient was operated of a L3–L4 lumbar spine stenosis by L3 laminectomy. A dural tear occurred during surgery, and the dural sac was stitched. Although his former symptoms regressed, he reported non-systematised pain in the lower limbs that he did not experience before. MRI shows clumped nerve roots, with no fluid between the roots and the dural sac (arrowheads) which is suggestive of adherences in an arachnoiditis. Sagittal (**a**) and axial (**b**) T2-weighted images

This corresponds to a non-infectious inflammation of the arachnoid with consequent adhesions between swollen nerve roots [11]. On T2-weighted images in the axial plane, intrathecal nerve roots are thickened, and can be clumped (pseudo mass) and/or adhere to the walls of the thecal sac, which results in an ‘empty sac’ [6, 11] (Fig. 8). Post gadolinium enhancement of the intrathecal roots and meningeal scarring may or may not be observed [11].

#### Radiculitis

This corresponds to an inflammation of the cauda equina nerve roots, diagnosed by a diffuse enhancement on post-contrast T1-weighted MR images [11]. Such images must be interpreted cautiously in the 6 months after surgery, as root enhancement can be seen in asymptomatic patients [7].

### Axial pain

#### Hardware failure: pseudarthrosis

In stabilisation-fusion procedures, intended to obtain joint immobilisation known as arthrodesis, fusion may fail, resulting in hardware loosening. Pseudarthrosis is defined as abnormal mobility in a level operated on that is supposed to be fused. Patients typically complain of lumbar pain recurrence after a pain-free interval of 3–6 months [20]. Radicular pain is uncommon. Although it can be diagnosed in the first year if fusion has never been achieved, pseudarthrosis can also happen up to a decade after surgery [20]. Diagnosing or ruling out pseudarthrosis is one of the most challenging situations in our experience and according to the literature [20].

##### Imaging modalities.

Plain radiographs and CT are the key combination in such situations [20]: plain radiographs allow baseline and follow-up examinations, as well as a dynamic evaluation [7, 14, 20], while CT is the method of choice for assessing bone fusion and loosening [8, 21]. Bone bridging is the key feature of a successful bone fusion [20]. In equivocal cases, in which mature bridging cannot be confidently determined, the nuclear medicine option can be helpful, by detecting increased bone metabolism at sites of motion [20]. Bone scintigraphy is the best option, but one must keep in mind two limitations: it lacks specificity, and radiotracer uptake related to bone healing and remodelling can persist up to 6–12 months after surgery [22]. MRI is not a good choice to assess bone fusion. The meaning of bone marrow oedema around expected fusion sites has been debated in the literature, but no clear consensus has emerged [20].

##### Expected findings in successful fusion.

**Fig. 9:**
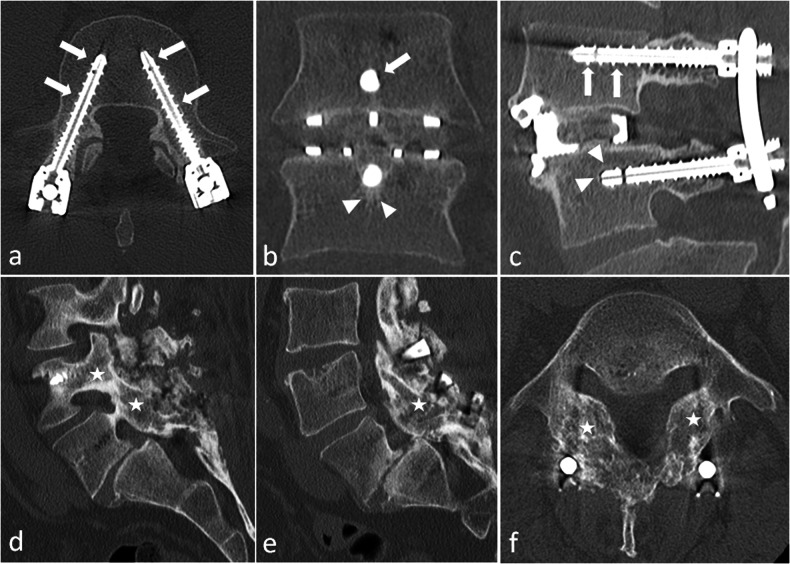
Normal aspect of an anterior and posterior lumbar intervertebral fusion, one year after surgery. On axial (**a**), coronal (**b**) and sagittal (**c**) CT images, no bone reaction, whether resorption or sclerosis, is seen around the screws (arrows), except a mild, thin sclerosis around the inferior screw on (**c**), which can be tolerated (arrowheads). Normal bone fusion in another patient. Sagittal (**d**, **e**) and axial (**f**) images show a full bony bridge on posterior columns, with cortical and trabecular continuity (stars). Also, no vacuum phenomenon (gas) is visible within the disks or around the interbody spacer

An early radiograph is taken to assess the initial position of screws, rods and implants to serve as a reference image [14, 15]. Bridging trabecular bone appears on radiographs between 3 months and 9 months after surgery [20]. At 12 months, trabeculation with strong cortical bone should be present between the vertebra [7, 20] (Fig. 9). No bone reaction is expected around the screws: a bone that is not exposed to direct mechanical constraints should not produce bone (Fig. 9). A regular, thin line of osteosclerosis and a regular, thin radiolucency can be tolerated. Cross-sectional imaging is generally not necessary in the first weeks or months. If it is done, one must pay attention not to overdescribe postoperative findings. Particularly, on MR images, substantial soft tissue oedema can be present, resulting in (asymptomatic) compression of the dural sac. On post-contrast images, cauda equina nerve roots can be enhanced in asymptomatic patients for up to 6 months after surgery [20].

##### Semiology of pseudarthrosis.

Pseudarthrosis is suspected in two situations.

**Fig. 10:**
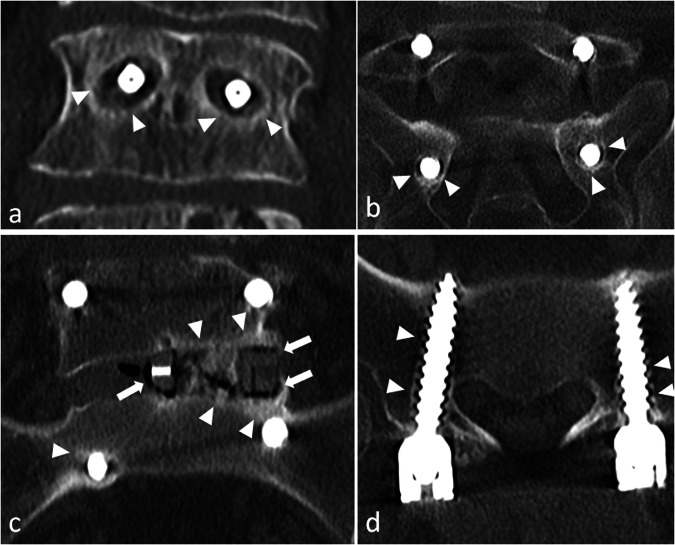
Typical aspects of mechanical pseudarthrosis in this patient who benefited from a posterior lumbar intervertebral fusion with an interbody spacer. Wide osteolysis with sclerotic margins realise the typical aspect of owl eyes on coronal CT images (arrowheads) (**a**). An identical aspect is seen all along the inferior screws (arrowheads) on coronal (**b**) and axial (**d**). Gas around and inside the interbody spacer (arrows) is the vacuum phenomenon secondary to micromobility. Osteosclerosis of the adjacent vertebral endplates (arrows **c**) and around the tip of one screw is also present because of this micromobility (**d**)

First, negative signs of successful fusion: lack of continuous bridging of trabecular bone in the expected time setting (1 year). That is to say, no bone bridge, or incomplete bridge, resembling enthesophytes of peripheral fibres in discarthrosis (Fig. 10).

Second, positive signs of micro-motion. The radiologist must search for traces of these movements on plain radiographs [14] and on CT, which is more accurate [8]. A very specific feature is the presence of gas in the disc or in the facet joints, which can be identified on CT images [20] (Fig. 10). In our experience, this is the best sign in difficult cases. As gas appears because of depression in the joint (cavitation phenomenon), its presence means that this intervertebral joint moves, which is not expected when fusion occurs. Although very suggestive of micromobility, the presence of gas is not mandatory, as hardware loosening can occur in the absence of gas. If gas has been identified, the radiologist must focus attention on this level and search for bone resorption around the screws and the intervertebral implant, if any (Fig. 10). This resorption is sometimes subtle and requires a three-dimensional analysis in the planes of each screw. Loosening is classically defined as bone resorption of more than 2 mm around the screw, predominating around the tip, and often surrounded by a thin sclerotic rim [20]. It gives the typical images of owl eyes in the coronal plane (Fig. 10). Inversely, wider, irregular bone resorption with ill-defined margins is highly suggestive of infection (Fig. 5).

Screw loosening occurs because of micro-motion. This situation is more frequent if screws are misplaced (Supplementary Fig. 7): a vicious circle begins, as loosening increases the micromovements that increase bone resorption around the screws [11]. Screws should ideally be centralised in the pedicles, with their extremity within the anterior part of the vertebral body [8].

Continuous biomechanical stress can result in hardware breakage. In posterior instrumentation, one must check for rod or screw breakage. This can occur if spine alignment has been incorrectly restored, resulting in mechanical constraints on rods, which can break. Plain radiographs are the modality of choice [14] as CT scans can be negative if rods are broken but not displaced (Supplementary Fig. 7). MR is not suitable. If gas is present in the intervertebral spaces but no abnormality is seen around the screw, a bone scan is needed to look for increased bone uptake around the screws (Supplementary Fig. 7).

Loosening of interbody spacers can be more difficult to diagnose. Indeed, the position of the spacer varies depending on the technique that was used and the local conditions during surgery. However, spacers should stand in the anterior part of the intervertebral space to restore lordosis, and should not break through vertebral endplates [6]. An interbody graft spacer is accurately positioned when the distance between the radiopaque marker of its posterior margin and the posterior vertebral body corner is 2 mm or greater [7]. Gas around and/or in the spacer can indicate micro-mobility, and other signs should be sought at the same level (Fig. 10). If gas is present only in a spacer, without any other sign of pseudarthrosis, this is the so-called ‘locked pseudarthrosis’, which is not considered a cause of instability [20]. Also, hardware-induced photon starvation can result in beam hardening artefacts, which is a differential diagnosis of the vacuum phenomenon in the disc. Modifying visualisation windows can make these artefacts appear more clearly, especially outside of the spacer.

**Fig. 11:**
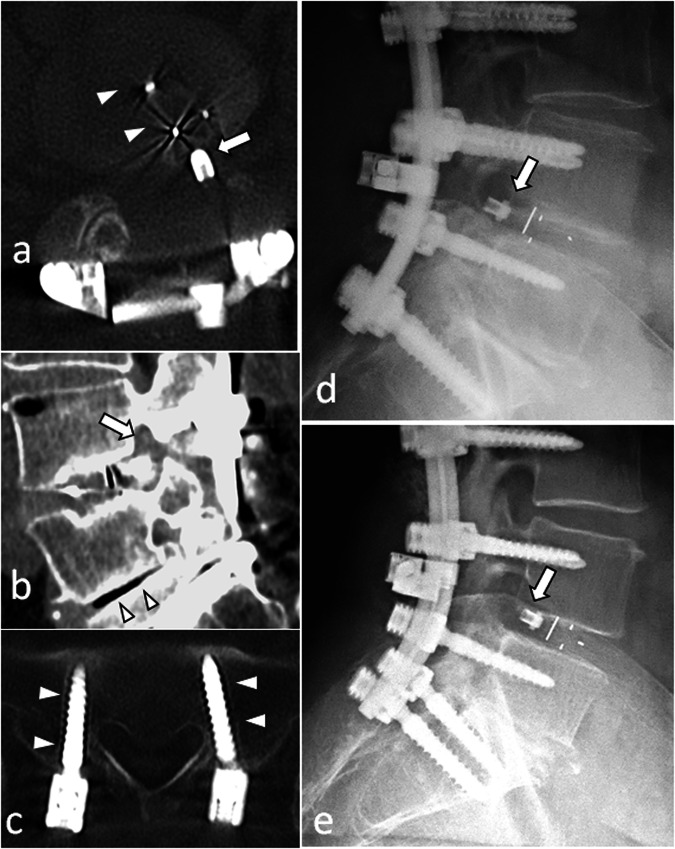
Patient referred for a left cruralgia that occurred after a 2 years pain-free interval following a lumbar arthrodesis with transforaminal lumbar interbody fusion (TLIF) at L4–L5 level. On axial CT-images with har kernel reconstructions (**a**) the interbody spacer (arrowheads), is lateralised, which is expected after TLIF surgery, but its posterior parts is seen beyond the disk limits (arrow). Sagittal soft kernel reconstruction (**b**) shows a narrowed right L4–L5 foramen, the spacer occupying the inferior part of the foramen. Conspicuous analysis reveals thin bone resorption around the S1 screws (arrowheads) (**c**). On contemporary lateral radiographs (**d**), the spacer seems too posterior. Comparison with baseline radiographs (**e**) confirms a posterior displacement of the spacer, which is diagnostic for pseudarthrodesis

Also, reference postoperative X-rays must be analysed and compared with current X-rays to check whether the spacer is in the same position (the posterior wall of adjacent vertebral bodies is a useful point of reference) [14]. If not, the spacer has moved, which means that the fusion has failed (Fig. 11). Of course, one must ensure that both X-rays are performed in the same conditions.

#### Adjacent segment syndrome

This term covers symptoms related to the development or progression of a mechanical pathology (disc, facets, and LSS, etc.) adjacent to a level operated on, or above or below it (Supplementary Fig. 8). It is caused by increased mechanical forces exerted on this hinge between the immobilised spinal level and adjacent spine segments [7]. The features of these pathologies are not distinguishable from those of patients not undergoing surgery. As with patients who have not had surgery, whether or not these pathologies are considered the source of pain depends on the imaging-clinical correlation, and imaging abnormalities are not always found, for example, in facet joint syndromes.

### Spine misalignment

**Fig. 12:**
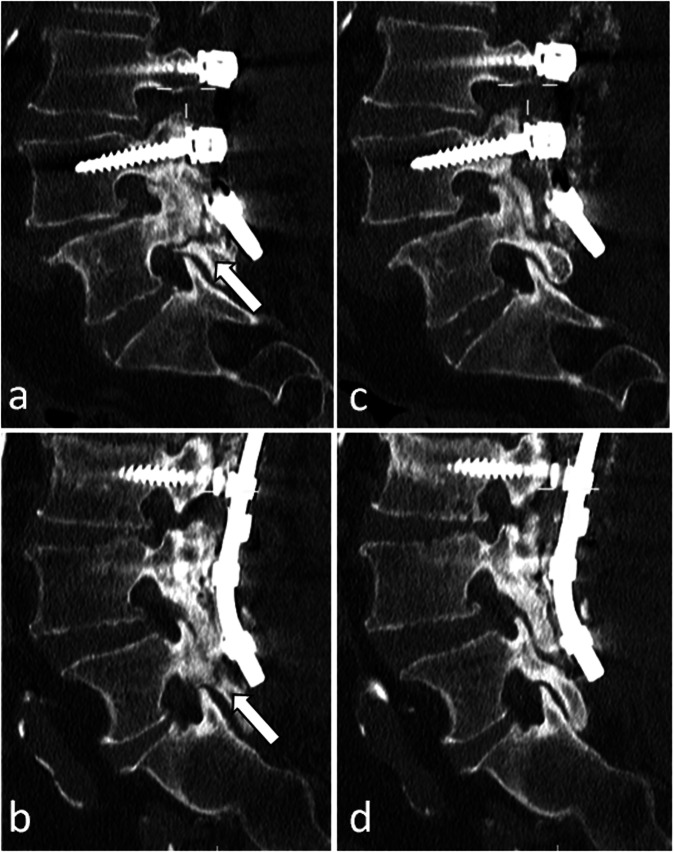
This patient complained of low back pain recurrence one year after a successful lumbar arthrodesis. On sagittal CT reconstructions (**a**, **b**) a bilateral pars articularis fracture is visible, which was unseen on a previous CT scan (**c**, **d**). This was attributed to an excessive stress on the posterior elements of the lumbosacral junction due to an incorrect restoration of the lordosis

Assessing spinopelvic alignment is crucial as surgery should restore a proper spine balance. Plain full-spine radiographs are the cornerstone of this assessment [14], looking for consistency between pelvic parameters (pelvic incidence, pelvic tilt, sacral slope) and spinal parameters (lumbar lordosis and thoracic kyphosis). Improper alignment correction can be one cause of pain: overcorrection can be a source of excessive stress on particular spine segments, leading to adjacent segment syndrome, while undercorrection may not be enough to relieve the stress on pathological elements [5] (Fig. 12 and Supplementary Fig. 7).

### Another pain … another problem?

If the patient complains of a type of pain that was never experienced before, then other conditions must be sought, as with patients not undergoing surgery. For example, vertebral fractures can occur in patients who have had surgery and those who have not, but also in the vicinity of surgical hardware [7].

## Conclusion

Postoperative spine imaging requires a good comprehension of the different surgical techniques and the potential inherent complications, knowledge of the precise symptoms and their timing, astute imaging modality choices, often comparing with former examinations, and a rigorous analysis of the images. Yet, even in these conditions, many situations can be very challenging: postoperative spine imaging is a lesson in humility. Everyone must keep in mind that patients can be psychologically fragile, with high expectations of their next imaging, especially in cases of chronic pain for which surgery can be considered as the solution to their suffering. Thus, conclusions should be cautious. Resolving difficult cases is rarely a single person’s responsibility. Listening to the patients, talking with the spine surgeons or other specialists (rehabilitation or rheumatologists), reading surgical reports, and browsing your picture archiving and communication system for former exams might be the key to offering the best chances of success.

## Supplementary information

ELECTRONIC SUPPLEMENTARY MATERIAL

## Data Availability

All data are available as figures.

## Abbreviations

CT: Computerised tomography
LSS: Lumbar spine stenosis
MRI: Magnetic resonance imaging
PET: Positron emission tomography
PSPS: Persistent spinal pain syndrome
SPECT: Single photon emission tomography

## Declarations

### Author information

J.-F.B. is a diagnostic and interventional musculoskeletal radiologist in Bretagne-Atlantique Hospital, and Professor of radiology at Lille Catholic University. T.M., J.L., and G.L. are diagnostic and interventional musculoskeletal radiologists. J.D. is a spine surgeon. V.D. is a rheumatologist. S.V. is Professor of radiology at Lille Catholic University, a neuroradiologist, and also head of the radiology department.
