# Supplementary material for: Imaging persistent spinal pain syndrome and spine surgery complications: an interpretation guide for radiologists
Source: Insights Imaging. 2026 Jan 28;17:26. doi: 10.1186/s13244-025-02065-8 (PMC12847616; doi:10.1186/s13244-025-02065-8)

**IMAGING PERSISTENT SPINAL PAIN SYNDROME AND SPINE SURGERY COMPLICATIONS:**

**AN INTERPRETATION GUIDE FOR RADIOLOGISTS**

**ELECTRONIC SUPPLEMENTARY MATERIAL**

**CLINICAL FEATURES**

**Acute cauda equina syndrome**

Cauda equina syndrome is defined by some or all of the following signs: urinary retention, urinary and/or faecal incontinence, saddle anaesthesia, pain in the back and/or legs and weakness or paralysis of usually more than one lumbar nerve root. It corresponds to a compression of the dural sac, which is likely caused by an epidural hematoma in early the postoperative context.

**Acute medullary syndrome**

It is suspected in case of pain associated with lower limb weakness and sensory troubles (numbness), and possibly from urinary retention, urinary and/or faecal incontinence. Key clinical elements are a sensory level (corresponding to the lower functional segment), motor deficits and hypo- or anaesthesia, and a pyramidal syndrome.

**Conus terminalis infarction**

Conus terminalis syndrome may associate sudden or acute back pain and radicular pain, motor and sensory deficits in the lower extremities, bladder dysfunction (retention or incontinence), bowel dysfunction (retention or incontinence), and saddle hypo- or a-nesthesia.

**Blood loss**

Postoperative haemorrhage usually occurs without external bleeding. According to blood loss volume, patients can be asymptomatic or experience abdominal and/or back pain. Tachycardia and polypnea are the first clinical signs, followed by a drop of the blood pressure. Haemoglobin concentration can be determined by rapid photometer tests such as HemoCue©, or by regular blood tests.

**Infection**

The incidence of postoperative infection in instrumented spines is 2-3% [1]. Early post-operative infection usually manifests by local pain, hyperthermia and elevated C-reactive protein. Yet this diagnosis can be clinically challenging because symptoms can overlap (surgery-induced back pain versus infection-related pain; differential diagnoses such as respiratory or urinary tract infections) [1].

**Post operative Headache**

Early postoperative headache is suggestive of intracranial hypotension secondary to a pseudomeningocele [2], which represents a fluid collection of cerebrospinal fluid that communicates with the intra-dural space but which is not confined by dura mater or a surrounding membrane [3]. Unlike meningoceles, the borders of a pseudomeningocele consist of reactive fibrous tissue [4]. Headaches are usually severe in intensity, with positional features, typically worsening in standing and sitting upright positions and also when bending the head forward, while relief occurs when lying down. Accompanying symptoms may occur with nausea, vomiting, dizziness, light-headedness, tinnitus, and blurred vision episodes.

**Pain-free interval**

The sensations of the patients during the first days and weeks after surgery can be difficult to interpret. Either with false improvement, because the patient limits its activities and adopts comfort positions (kinesiophobia), or takes unusual (often stronger) painkillers. Or contrarily with false deterioration, because the patient feels pain that is related to the surgical procedure, secondary to the inflammation of the surgical bed, which is expected as it is a physiological healing phenomenon.

After a period of several weeks, the patient can be assessed more reliably. In this chapter we will consider that the patient does not report a significant change in the symptoms after surgery. In the next chapter, we will consider another situation, where the patients feel significantly better during several weeks, months or years, and reports a recurrence of the same symptoms or a different kind of pain.

**Technically perfect treatment, but no improvement**

If the postoperative imaging shows a technically-successful procedure in a patient that does not report any clinical improvement, we advise to consider a differential diagnosis: has something been missed? For example, in case of a totally unsuccessful treatment of a nerve root decompression, the issue of referred pain needs to be discussed, as facet joint syndromes can be responsible for back pain radiating in the leg [5]. Of course, this is supposed to be done before surgery, but cognitive bias such as anchoring bias may impede the consideration of differential diagnoses, and lead to inappropriate decisions [6]. This discussion is beyond the scope of this article, but curious reader can learn more on this subject in the article written by Zhang et al., recently published in Insights Into Imaging [6].

If no differential diagnosis seems valuable, the expert advice of pain medicine specialists can be sought, as chronic low back pain is a multifactorial condition, that can find its roots far beyond anatomical conditions and imaging descriptions [7].

1. Expert Panel on Neurological Imaging, Ortiz AO, Levitt A, et al (2021) ACR Appropriateness Criteria® Suspected Spine Infection. J Am Coll Radiol JACR 18:S488–S501. https://doi.org/10.1016/j.jacr.2021.09.001

2. Eisenmenger L, Clark AJ, Shah VN (2019) Postoperative Spine: What the Surgeon Wants to Know. Radiol Clin North Am 57:415–438. https://doi.org/10.1016/j.rcl.2018.10.003

3. Petscavage-Thomas J, Ouyang T, Bible J (2020) Spine Fixation Hardware: An Update. AJR Am J Roentgenol 215:534–544. https://doi.org/10.2214/AJR.20.22810

4. Ghodasara N, Yi PH, Clark K, et al (2019) Postoperative Spinal CT: What the Radiologist Needs to Know. Radiogr Rev Publ Radiol Soc N Am Inc 39:1840–1861. https://doi.org/10.1148/rg.2019190050

5. Perolat R, Kastler A, Nicot B, et al (2018) Facet joint syndrome: from diagnosis to interventional management. Insights Imaging 9:773–789. https://doi.org/10.1007/s13244-018-0638-x

6. Zhang L, Wen X, Li J-W, et al (2023) Diagnostic error and bias in the department of radiology: a pictorial essay. Insights Imaging 14:163. https://doi.org/10.1186/s13244-023-01521-7

7. Chan C, Peng P (2011) Failed Back Surgery Syndrome. Pain Med 12:577–606. https://doi.org/10.1111/j.1526-4637.2011.01089.x

**SUPPLEMENTARY FIGURES**

Supplementary figure 1

(a, b) Postoperative venous bleeding in a patient with subacute deglobulisation after spine surgery. Axial non-contrast CT scan. The bleeding (arrows) is in the subcutaneous tissues.

(c) Differential diagnosis of a venous bleed: subcutaneous oedema (arrowheads). The distribution of the hypodense infiltration is diffuse, and the context is different as this patient remained lying for days in an intensive care unit. Note the presence of ascites (asterisks).

(d) Haemoperitoneum. CT scan was performed for deglobulisation after L4-L5 herniated disc treatment, showing a voluminous haemoperitoneum visible on the non-contrast acquisition (arrows) caused by an internal iliac artery injury.


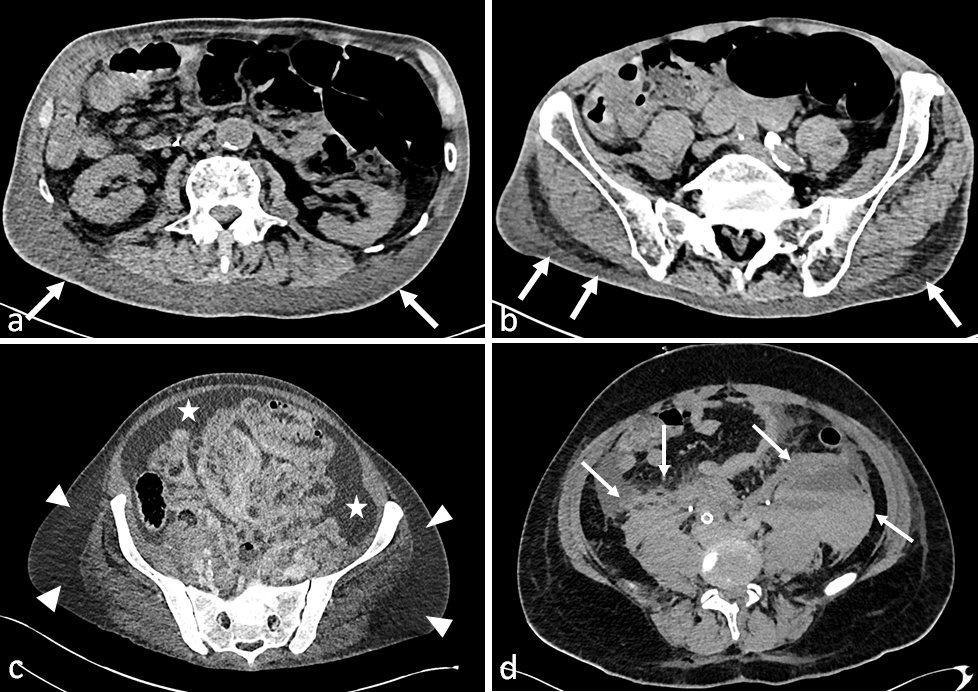


Supplementary figure 2

(a, b) Immediate postoperative right S1 radicular pain after lumbosacral posterior arthrodesis. CT reveals a screw malposition (arrow) within the right first sacral foramen on coronal (a) and sagittal (b) reconstructions. (c, d) Another patient woke up with right thigh pain after oblique lumbar interbody fusion (OLIF) arthrodesis. The CT scan shows an oversized implant extending beyond the vertebral body level into the right L4-L5 foramen (arrow), therefore conflictual with the right L4 nerve root, which is swollen (arrow). (e-g) A third patient had an MRI due to posterior right thigh pain immediately after L4-S1 arthrodesis. On coronal T2-weighted images (e) a conflict between the L5 nerve root (arrowheads) and an extrapedicular L5 screw (arrow) was diagnosed by a junior radiologist. After senior expertise, a CT scan was performed, confirming the extrapedicular position of the screw (arrow), yet without contact with the right L5 root (arrowheads) on the coronal (f) and sagittal (g) reconstructions, as the structures are separated by epidural fat.
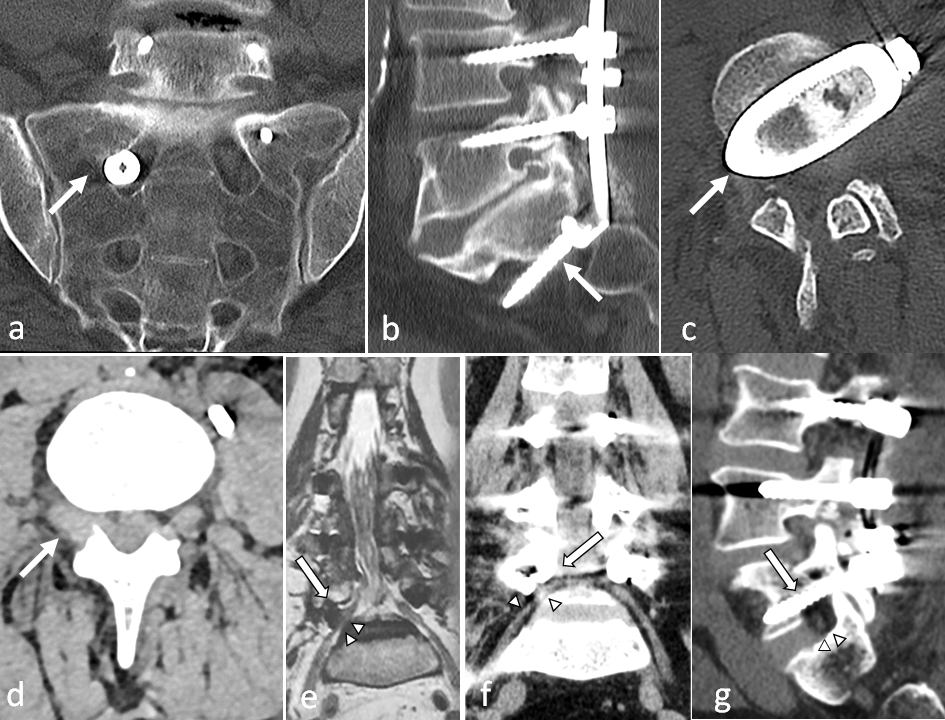


Supplementary figure 3

(a-d) Patient with left L5 radicular pain who underwent surgery for L4-L5 paramedian disc herniation by endoscopic transforaminal L4-L5 approach. The patient reported immediate postoperative previously unknown anterior left thigh pain. On axial (a) and coronal (b) CT scan reconstructions, the left L4 root is swollen (arrowheads) in comparison with the contralateral root (arrow). On sagittal (c) and axial (d) post-contrast T1-weighted MR images with fat suppression, the L4 root enhances (arrowhead), whereas L3 and L5 roots (arrows, c) and right L4 root (arrow, d) do not. This corresponds to a right L4 battered nerve. (e, f) In another patient complaining of diffuse left leg pain a few days after anterior lumbar interbody fusion (ALIF), a post-contrast CT scan shows deep vein thrombosis of the left common iliac vein (arrows) on axial (e) and coronal (f) reconstructions.


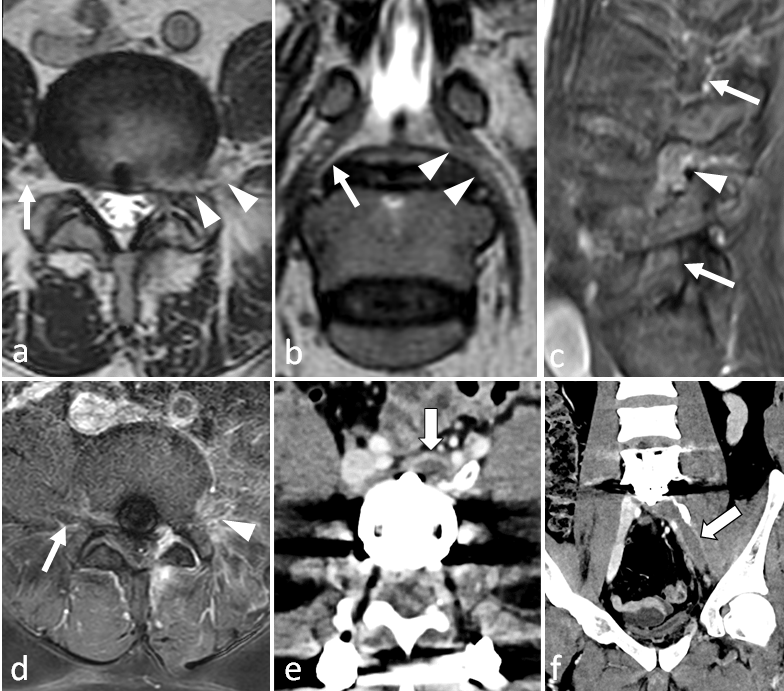


Supplementary figure 4

(a-c) Seroma in an asymptomatic patient treated for disc herniation: posterior fluid collection on the operative path that appears hypointense on post-contrast T1-weighted images (a), hyperintense on T2-weighted images (b) and hypointense on sagittal post-contrast fat-suppressed T1-weighted images (c). Note the peripheral enhancement (arrowheads) that corresponds to a physiological inflammatory reaction. (d, e) Postoperative MRI in a patient treated for lumbar spine stenosis with a peroperative dural tear that was fixed with surgical glue. Glue appears mildly hypointense on T2-weighted images (d), and clearly hypointense on post-contrast T1-weighted images (e), amid an ill-defined infiltration of the surgical bed that is hyperintense on T2-weighted images (d) and that enhances on post-contrast images (arrows) (e). (f-h) Normal appearance of a bone harvesting site in the posterior iliac bone (asterisks) (f, g) with gas (arrowhead) that must not be mistaken for an abscess. In this patient, the hydroaeric content extends upwards along the paravertebral muscles (arrows).


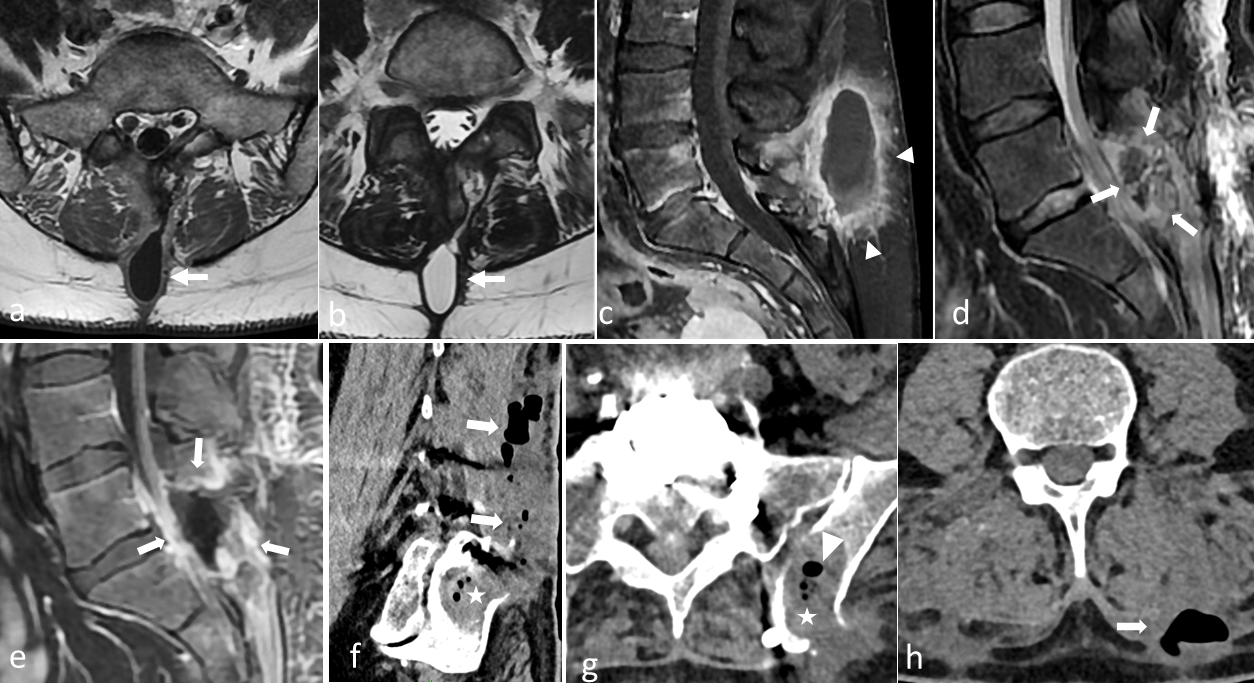


Supplementary figure 5

(a) On this postoperative MRI T2-weighted image of an L3-L4 lumbar spine stenosis, the dural sac compression is still present (arrows) whereas the dural sac is expanded at L4-L5 level (arrowheads), which corresponds to a level error. (b) On another patient treated for multilevel lumbar spine stenosis, MRI sagittal T2-weighted images show an insufficient decompression: laminectomy was performed correctly in L2 and L3 (arrows), but not in L4, which resulted in a persistent L4-L5 stenosis (arrowheads). (b, c) A third patient was operated on for an L3-L4 hernia (asterisk) responsible for lumbar spine stenosis (arrowhead): preoperative MRI with sagittal T2-weighted images (c). On the postoperative CT scan, the herniation is still present (arrow), and the surgical path is visible at the level above (arrows), which corresponds to a level error.


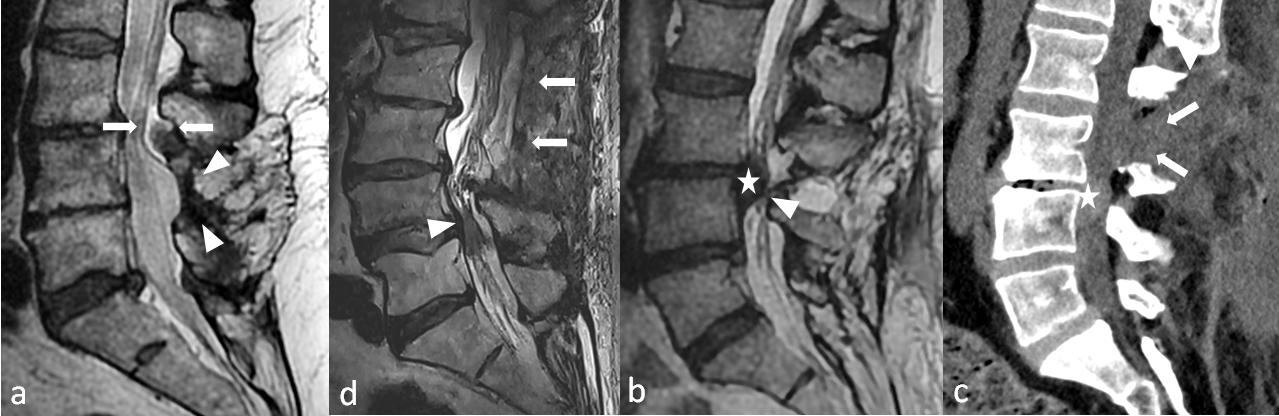


Supplementary figure 6

(a-c) Normal postoperative status 1 month after a herniectomy. On preoperative axial T2-weighted MR image (a), a central protrusion (arrows) is in conflict with the nerve roots in the lateral recesses (arrowheads). On postoperative images, the surgical bed appears as a mildly hyperintense heterogeneity of the anterior and right epidural space (arrowhead) on axial MRI T2-weighted images (b) that continues through the ligamentum flavum and along the spinous process (arrows). On post-contrast fat-suppressed T1-weighted images (c), this recent scar enhances strongly (arrowheads) and is swollen, which tends to collapse the emerging root (arrow). (d, e) In another paient, normal postoperative status 6 months after a herniectomy: the scar is more mature than the previous example. On axial T2-weighted MR images (d), it is hypointense (arrowheads) and retractile, which results in an attraction of the dural sac (thick arrow) and an enlargement of the dural expansion of the emerging root (arrows). On the post-contrast T1-weighted image (e), the scar enhances (arrowheads). In this case, note the associated enhancement of the facet joint capsule (asterisk). (f, g) In a last patient presenting with recurrent radicular pain in the same territory 2 months after surgery, the comparison of preoperative (f) and postoperative (g) axial T2-weighted images shows a disc herniation in the same topography, but with a different morphology (arrows and arrowhead), which is diagnostic for hernia recurrence. As morphological images answer the question, a gadolinium injection is not necessary.


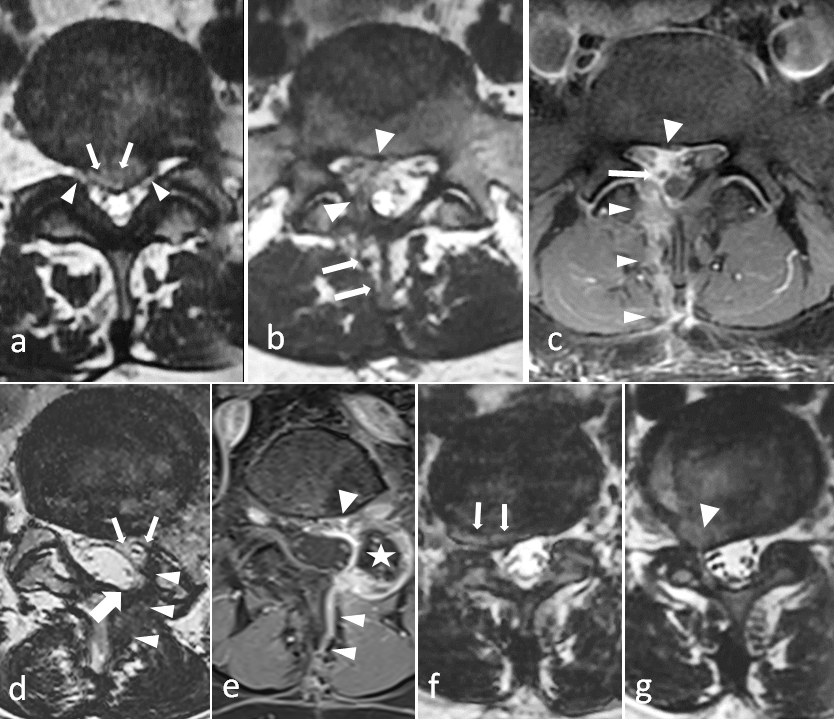


Supplementary figure 7

(a-e) CT scan performed one year after thoracolumbar arthrodesis in a patient presenting with pain recurrence after a pain-free interval. On thick MPR reconstructions in the sagittal (a, b) and coronal (c) planes, many lumbar screws begin their course within the pedicles, but end in the vertebral endplates or even in the discs (arrowheads). However, no clear bony resorption is seen around the screws. A conspicuous analysis of axial images (d) and posterior structures reveals a mild bone resorption around a screw (arrows). Also, facet joints (asterisks) are still visible at the same level amid the albeit posterior fused columns, although they should be fused. Bone scintigraphy (e) shows increased tracer uptake around some screws, which confirms micromobility and thus pseudarthrodesis. (f-j) This other patient reported upper lumbar pain 18 months after a long thoracolumbar arthrodesis. Sagittal CT images (f-h) reveal a clear multilevel intradiscal vacuum (arrowheads) reflecting mobility and thus pseudarthrodesis. The analysis of posterior structures shows cracks within the posterior columns (arrows) (f, g). Rod breakage is clearly visible on one side (thick arrow) (g), but subtler on the other (double arrow) (h), as there is little space between the two parts of the broken rod. This is far clearer on radiographs (arrows) (I, j) as this technique does not suffer from beam hardening artifacts. Note the marked kyphosis on the lateral view (j), which suggests an incorrect surgical correction of the sagittal balance with exaggerated mechanical stress on the rods.


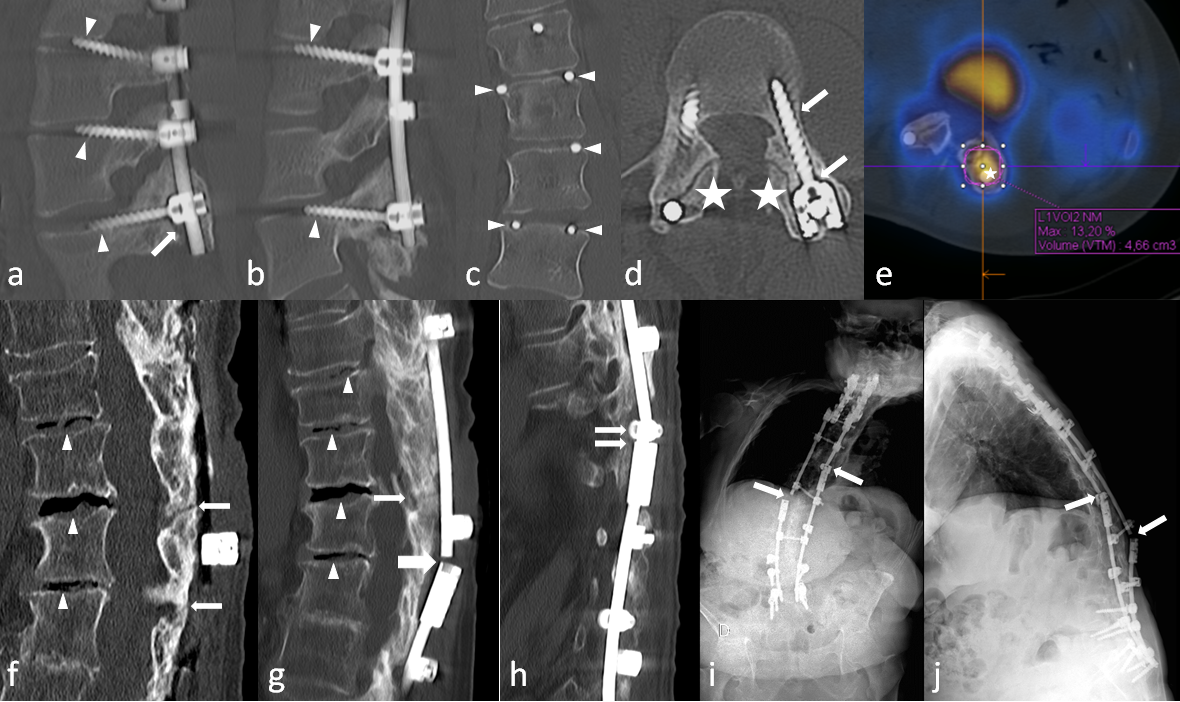


Supplementary figure 8

Adjacent level syndrome in a patient who underwent a L4-L5 laminectomy for lumbar spine stenosis 10 years ago, who complained of pluriradicular pain while walking. The benefit of surgery is still present in L4-L5 where the dural sac is wide (asterisk). On T2-weighted sagittal images (a), the dural sac is narrowed at the L2-L3 and L3-L4 levels due to disc bulging (arrows) and epidural fat bulging (arrowheads) visible. Axial images (b) show the disc (arrows) and the ligamentum flavum bulging (arrowheads).


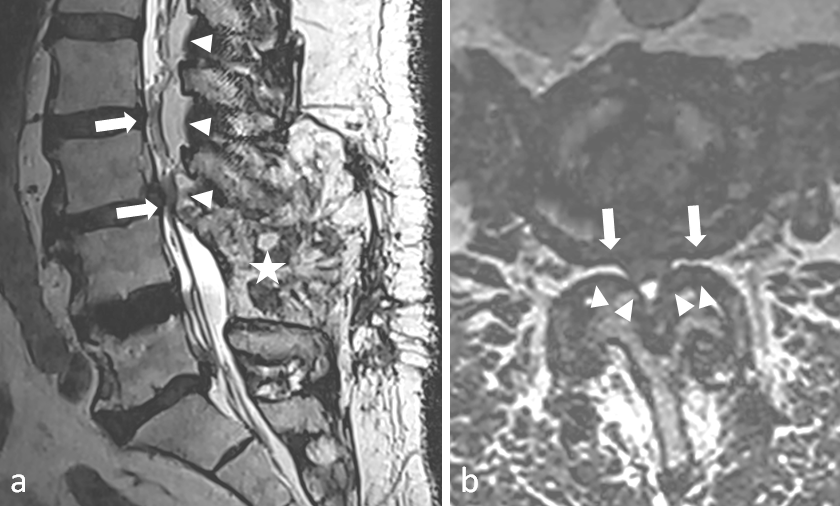

Supplement: Supplementary file 1 — ELECTRONIC SUPPLEMENTARY MATERIAL [file 13244_2025_2065_MOESM1_ESM.docx]
